# Deutschlandweite Befragung der Lehrbeauftragten in der Augenheilkunde zur studentischen Lehre in der Corona-Pandemie 2020/21

**DOI:** 10.1007/s00347-021-01544-9

**Published:** 2021-12-09

**Authors:** Anna L. Engel, Andreas Müller, Helene Spät, Sandra Kurz, Esther M. Hoffmann

**Affiliations:** 1 Augenklinik und Poliklinik, Universitätsmedizin Mainz, Johannes Gutenberg-Universität, Langenbeckstr. 1, 55131 Mainz, Deutschland; 2 Augenärzte Dr. Jaksche und Kollegen, Ansbach, Deutschland; 3 Anästhesiologie und Rudolf Frey Lernklinik, Universitätsmedizin, Johannes Gutenberg-Universität, Mainz, Deutschland

**Keywords:** Augenheilkunde, Medizinische Lehre, „Blended learning“, COVID-19, Hybride Lehre, Ophthalmology, Medical education, Blended learning, COVID-19, Hybrid teaching

## Abstract

**Hintergrund:**

Die Corona-Pandemie hat einen erheblichen Einfluss auf die Bedingungen der universitären studentischen Lehre. Durch die pandemiebedingten Kontaktbeschränkungen kamen vielerorts digitale Lehrformate anstatt der bisherigen Präsenzlehre zum Einsatz. Diese wurde im Sommersemester 2020 durch die Studierenden in bisher vorliegenden Veröffentlichungen teils schon gut angenommen und positiv evaluiert. In dieser Arbeit wurde das Hauptaugenmerk auf die Erfahrungen und Einschätzungen der Lehrenden in der Augenheilkunde während des Wintersemesters 2020/21 gelegt.

**Methodik:**

Anhand zweier anonymisierter Befragungen mittels Online-Fragebögen wurden zum einen die Lehrbeauftragten der Augenheilkunde der deutschen Universitätskliniken sowie zum anderen interne und externe Dozierende und Mitarbeitende in der studentischen Lehre der Augenklinik der Universitätsmedizin Mainz zu ihren Erfahrungen mit der Implementierung der digitalen Lehre befragt.

**Ergebnisse:**

Hierbei gaben 95 % der Lehrbeauftragten der Augenkliniken der Universitätskliniken in Deutschland an, spätestens seit der Corona-Pandemie digitale Lehrkonzepte etabliert zu haben. Bei 68 % kamen Hybridformate mit anteiliger Präsenzlehre zum Einsatz. Es wurden vielfältige Lehrformate angewendet. Hierbei traten auch Schwierigkeiten, insbesondere in der Interaktion mit den Studierenden sowie durch unzureichende technische Ausstattung der Kliniken, auf. Trotz überwiegend digitaler Lehre wurden weiterhin erprobte Prüfungskonzepte in Präsenzform angewendet, nur 18 % der Befragten gaben an, Online-Prüfungen durchgeführt zu haben. Künftig wollen 86 % der Befragten digitale Formate in ihre Lehrkonzepte integrieren und als Ergänzung der bisherigen Präsenzlehre etablieren.

**Diskussion:**

Die Entwicklung der studentischen Lehre während der Corona-Pandemie kann als Chance für die Gestaltung der zukünftigen Ausbildung von Medizinstudierenden in der Augenheilkunde dienen.

## Abstract (en)

**Background:**

The corona pandemic has had a significant impact on the conditions of university student teaching. Due to the pandemic-related contact restrictions, digital teaching formats were widely used instead of the previous face-to-face teaching. In the summer semester of 2020 students received this well and evaluated it positively in recent publications. In this work, the main focus was on the experiences and assessments of teachers in ophthalmology during the winter semester 2020/2021.

**Methods:**

By means of two anonymous surveys via online questionnaires, the lecturers in ophthalmology of German university hospitals as well as internal and external lecturers and staff members of the student teaching of the Department of Ophthalmology of the University Medical Center Mainz were asked about their experiences with the implementation of digital teaching.

**Results:**

In this context 95% of the teaching staff of ophthalmology departments of university hospitals in Germany stated that they had established digital teaching concepts at the latest since the corona pandemic. Hybrid formats with a proportion of face-to-face teaching were used by 68%. A wide variety of teaching formats were used. Difficulties were also encountered, particularly in interaction with students. Despite predominantly digital teaching, examinations continued to be held in face-to-face settings; only 18% of respondents stated that they had conducted online examinations. In the future, 86% of respondents want to integrate digital formats into their teaching concepts and establish them as a supplement to existing face-to-face teaching.

**Conclusion:**

The development of student teaching during the corona pandemic can serve as an opportunity for shaping the future education of medical students in ophthalmology.

## Hintergrund

Die Corona-Pandemie brachte uns alle im letzten Jahr in eine beispiellose Ausgangslage. Durch pandemiebedingte Kontaktbeschränkungen sind zahlreiche Umstellungen der studentischen Lehre an den Universitätskliniken nötig geworden, auch in der studentischen Lehre in der Augenheilkunde. Dies verlangte zwar ein großes Engagement der Lehrenden, ermöglichte aber gleichzeitig eine Basis für die Gestaltung des digitalen Wandels in Lehre und Studium [4]. Die Präsenzlehre und klinische Ausbildung am Patienten wurde aufgrund der Hygienevorgaben und -auflagen deutlich eingeschränkt. Dabei kamen stattdessen vielfältige digitale Lehrformate zum Einsatz. Diese waren zum Teil (z. T.) schon vor dem Beginn der pandemiebedingten Veränderungen etabliert worden oder in der Erprobung. Einige Formate wurden jedoch erst durch die neuen Vorgaben zum Infektionsschutz fest in die Lehrpläne eingearbeitet.

An der Augenklinik der Universitätsmedizin Mainz wurde als Reaktion auf die coronabedingten Kontaktbeschränkungen das Konzept der studentischen Lehre angepasst. So wurden für das 5. und 6. Semester Vorlesungen durch Vorlesungspodcasts ersetzt. Anstatt des praktischen Untersuchungskurses im 5. Semester wurden Untersuchungsvideos und ein Online-Tutorium zur Verfügung gestellt. Die Präsenzlehre des 6. Semesters, welche zuvor aus Hospitation in der Ambulanz, im Operationssaal und der selbstständigen Durchführung von Anamnese und Spaltlampenuntersuchung bestand, wurde durch digitale Angebote ersetzt. Dabei wurden in einem Live-Patientenzimmer per Video-Spaltlampe Anamnese und Untersuchung von Patienten per Videostream live demonstriert. Über eine Chatfunktion war dabei parallel eine Interaktion der Studierenden und Lehrenden möglich. Zudem wurden anonymisierte Anamnese- und Operationsvideos mit Erklärungen zur Verfügung gestellt. Anhand interaktiver Patientenfälle konnten wichtige „red flags“ der Augenheilkunde eingeübt werden. Die Evaluation durch die Studierenden zu den digitalen Lehrkonzepten war sehr positiv. Der digitale Unterricht der Augenklinik Mainz wurde im Sommersemester 2020 im 5. Semester im Mittel mit einer Schulnote von 1,58 (Standardabweichung 0,75) bewertet, im 6. Semester mit einer Schulnote von 2,18 (Standardabweichung 1,07) [8]. Insbesondere das virtuelle Patientenzimmer (Mittelwert 1,46, Standardabweichung 0,74), die interaktiven Patientenfälle (Mittelwert 1,51, Standardabweichung 0,68) und die Videos zu Untersuchungstechniken (Mittelwert 1,42, Standardabweichung 0,64), Anamnese (Mittelwert 1,92, Standardabweichung 0,9) und Operationen (Mittelwert 2,13, Standardabweichung 0,87) wurden gut bewertet. Der Erwerb praktischer Kompetenzen konnte digital allerdings nur teilweise realisiert werden. Im hybriden Wintersemester 2020/21 wurde deshalb eine Kombination der digitalen Lehrformate und eines Präsenzunterrichts in Kleingruppen durchgeführt [8].

Andere Kliniken in Deutschland kamen zu ähnlichen Ergebnissen. Befragungen zur Einschätzung der digitalen Lehrformate während der Corona-Pandemie aus studentischer Sicht zeigten z. B. an der Augenklinik des Universitätsklinikums Lübeck, dass Studierende die digitalen Lehrformate gut annehmen und sich auch für die Zukunft eine Ergänzung der bisherigen Lehrpläne um solche Formate vorstellen können. In der Erhebung aus Lübeck wurde sogar eine höhere Teilnahmerate an den Live-Online-Vorlesungen registriert als bei den vorherigen Präsenzvorlesungen. Dabei wurde von den Studierenden angemerkt, dass eine gute Plattform zur Kommunikation und dem Austausch sowohl mit den Dozierenden als auch mit den Kommilitonen als sehr wichtig eingeschätzt wurde. Negativ bewertet wurde von den Studierenden erwartungsgemäß v. a. das Fehlen der praktischen Übungen, welche insbesondere in der Augenheilkunde einen hohen Stellenwert haben [7]. Auch in einer Erhebung aus den USA gaben Medizinstudierende an, dass fehlender Kontakt mit der Augenheilkunde einer der Hauptgründe sei, weshalb sie sich gegen die Ausbildung zum Augenarzt entschieden [6]. Ziel muss also – trotz der pandemiebedingten Einschränkungen – sein, die Medizinstudierenden und damit potenziellen zukünftigen Kolleginnen und Kollegen für die Augenheilkunde zu begeistern. Ein Weg dafür ist eine ansprechende und zukunftsfähige Gestaltung der augenheilkundlichen Lehre.

Unsere Erhebung hatte deshalb das Ziel, die Erfahrungen und Einschätzungen der Lehrbeauftragten der universitären Augenkliniken in Deutschland zur studentischen Lehre während der Corona-Pandemie und bezüglich möglicher zukünftiger Lehrformate zu untersuchen.

## Methodik

Es wurden die Lehrbeauftragten der Augenheilkunde der 37 deutschen Universitätskliniken kontaktiert. Sie bzw. ihre maßgeblich an der studentischen Lehre beteiligten ärztlichen Mitarbeiter wurden via einer anonymisierten Online-Umfrage zu ihrer Einschätzung zur Situation im Corona-Semester Wintersemester 2020/21 befragt. Es nahmen 34 Personen im Zeitraum März bis Mai 2021 an der Umfrage teil, 22 von 34 (65 %) beantworteten den Fragebogen vollständig. Es wurden in 20 Fragen (Mehrfachauswahl, Listenoption, Freitextantworten) die vor der Corona-Pandemie und aktuell zur Anwendung kommenden Lehr- und Prüfungsformate sowie die persönliche Einschätzung von Vorteilen und Schwierigkeiten der digitalen Lehrformate erfasst. Dabei wurden jeweils alle registrierten Antworten in der Auswertung berücksichtigt.

In einer zweiten anonymisierten Online-Umfrage wurden die als Tutoren und Dozenten an der studentischen Lehre in der Mainzer Augenklinik beteiligten ärztlichen Mitarbeiter zu ihrer Einschätzung zur Situation im Corona-Semester Wintersemester 2020/21 befragt (davon waren 7 Befragte habilitiert, 3 nicht habilitierte Fachärzte und 7 Assistenzärzte der Mainzer Augenklinik sowie 6 externe Dozierende). Es nahmen 20 von 23 (87 %) befragten lehrenden, ärztlichen Mitarbeitern an der Umfrage teil, 14 von 23 (61 %) beantworteten den Fragebogen vollständig. Es wurden ebenfalls jeweils alle registrierten Antworten in der Auswertung berücksichtigt.

## Ergebnisse

### Nationale Befragung

In der deutschlandweiten Befragung unter den Lehrbeauftragten der Augenheilkunde der Universitätskliniken (*n* = 22) gaben 96 % an, im Wintersemester 2020/21 online augenheilkundliche Lehrveranstaltungen angeboten zu haben, bei 68 % der Teilnehmenden haben Präsenzveranstaltungen stattgefunden.

Vor der pandemiebedingten Umstellung der Lehrkonzepte waren die Unterrichtsveranstaltungen in der Augenheilkunde wie folgt (Tab. 1):

**Tab. 1:** Darstellung der Lehrformate vor der Corona-Pandemie

Unterrichtsveranstaltungen vor der Corona-Pandemie	Häufigkeit in Prozent (absoluter Wert)
Praktikum	96 (*n* = 21)
Vorlesung	96 (*n* = 21)
Untersuchungskurs (Augenspiegelkurs)	82 (*n* = 18)
Operationskurse/„wet labs“	50 (*n* = 11)
Sonstige (z. B. Seminare, Problemorientiertes Lernen, Wahlfächer)	46 (*n* = 10)

In unserer Befragung gaben 41 % (*n* = 9) an, seit dem WiSe 19/20 digitale Lehre zu nutzen, 59 % (*n* = 13) bereits seit dem Sommersemester (SoSe) 2020.

Dabei kamen verschiedene digitale Lehrformate wie interaktive Online-Videoseminare, aufgezeichnete Vorlesungen und aufgezeichnete Untersuchungsvideos zum Einsatz, zukünftig wollen 86 % (*n* = 19) digitale Lehre in ihr reguläres Lehrkonzept implementieren (Tab. 2).

**Tab. 2:** Verwendung digitaler Lehrformate im Wintersemester 2020/21 und die zukünftig geplante Verwendung

Digitale Lehrformate	Verwendung *n* = 22 Fragebögen, Häufigkeit der Antwort in Prozent (Anzahl)	
	Im WiSe 20/21	Zukünftig geplant
*Online-Videoseminare*		
Interaktiv	86 (*n* = 19)	55 (*n* = 12)
Nicht-interaktiv	23 (*n* = 5)	9 (*n* = 2)
*Livestreams von Vorlesungen*	32 (*n* = 7)	27 (*n* = 6)
*Aufgezeichnete Vorlesungen*		
Ohne Interaktion	86 (*n* = 19)	36 (*n* = 8)
Mit Interaktionsmöglichkeit (z. B. Quizfragen)	23 (*n* = 5)	36 (*n* = 8)
*Aufgezeichnete Videos zu unterschiedlichen Techniken und Vorgängen (Spaltlampenuntersuchung, Konfrontationsperimetrie etc.)*	68 (*n* = 15)	59 (*n* = 13)
*Live-Patientendemonstration im Rahmen eines „virtuellen Patientenzimmers“*	14 (*n* = 3)	18 (*n* = 4)
*Sonstige (z.* *B. digitales Blockpraktikum, Online-Arbeitsblätter, Repetitoriumsfragen, Wiener Augenfälle)*	23 (*n* = 5)	5 (*n* = 1)

**Abb. 1:**
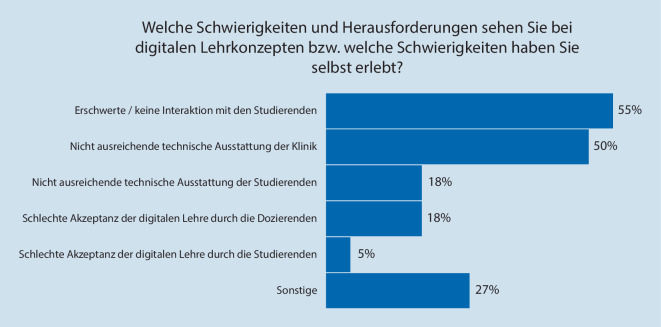
Darstellung der Einschätzung zu Schwierigkeiten bei digitalen Lehrkonzepten in der deutschlandweiten Befragung

Befragt zu den Schwierigkeiten bei digitalen Lehrkonzepten, gaben 55 % (*n* = 12) der Lehrbeauftragten an, dass die Interaktion mit den Studierenden erschwert sei. Weitere 50 % (*n* = 11) gaben an, dass ihre Klinik technisch nicht ausreichend ausgestattet sei. Die technische Ausstattung der Studierenden wurde nur von 18 % (*n* = 4) der Umfrageteilnehmer als Schwierigkeit gesehen, ebenso wurde von 18 % (*n* = 4) die Akzeptanz der digitalen Lehre durch die Dozierenden als schwierig eingeschätzt. Die Akzeptanz der Lehre durch die Studierenden hingegen sah nur 1 Teilnehmer als Herausforderung. Bei weiteren 27 % (*n* = 6) seien sonstige Schwierigkeiten aufgetreten (z. B. ein initial erhöhter Aufwand und die schwierige Vermittlung praktischer Fähigkeiten) (Abb. 1).

**Abb. 2:**
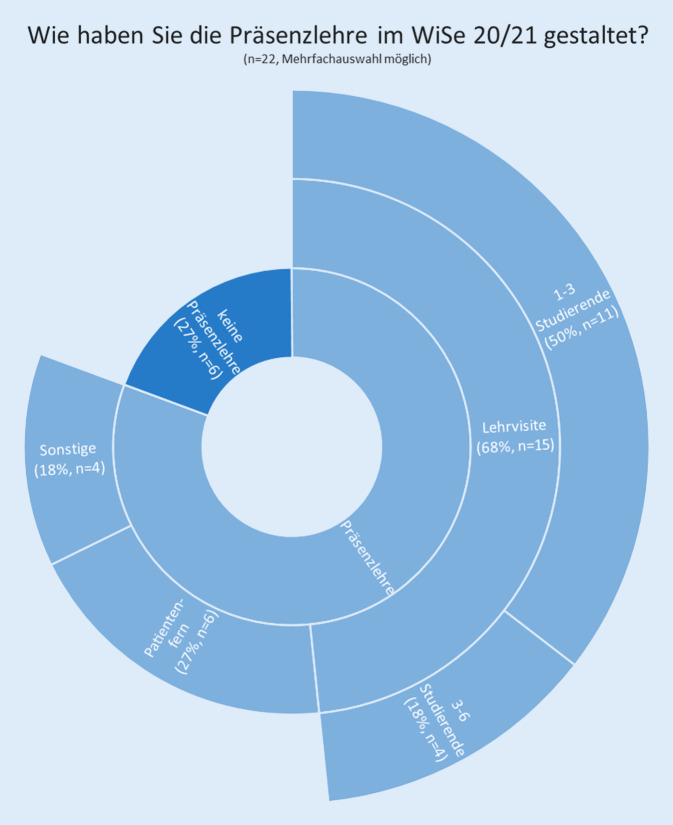
Darstellung der Angaben zur Präsenzlehre im Wintersemester 2020/21 in der deutschlandweiten Befragung

Wir befragten die Lehrbeauftragten der deutschen universitären Augenkliniken auch zur Durchführung von Präsenzlehre im Wintersemester 2020/21 (Abb. 2). Dabei gaben 27 % (*n* = 6) an, keine Präsenzlehre durchgeführt zu haben. Wenn Präsenzlehre angeboten wurde, kamen Lehrvisiten und patientenferner Unterricht in Kleingruppen sowie sonstige Formate (z. B. an Op.-Simulatoren oder Op.-Hospitationen) zum Einsatz. Insgesamt fand jeweils bei 50 % (*n* = 11) der Befragten Unterricht mit Patientenkontakt und bei 50 % (*n* = 11) der Befragten Unterricht ohne Patientenkontakt statt. Bei 86 % (*n* = 19) gab es zentral festgelegte Hygienekonzepte für den Unterricht am Patienten.

**Abb. 3:**
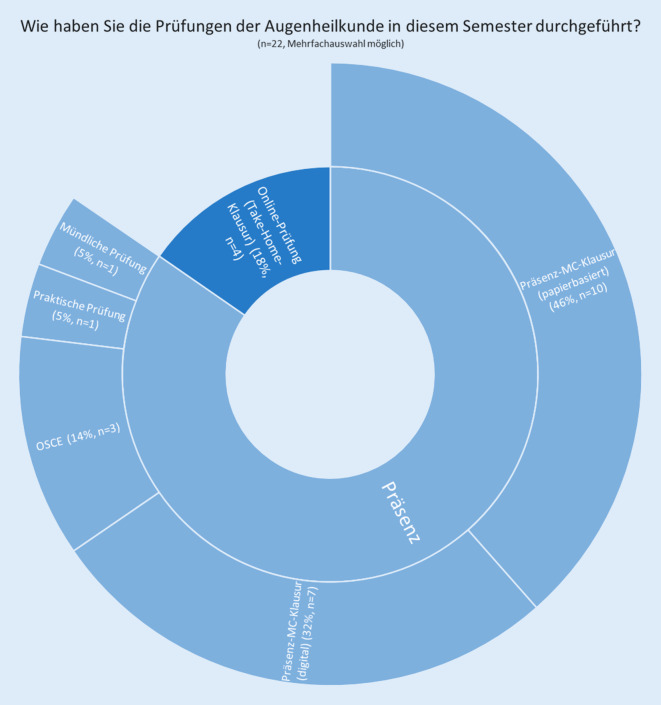
Darstellung der Angaben zur Durchführung von Prüfungen in der deutschlandweiten Befragung (*MC* Multiple Choice, *OSCE* Objective Structured Clinical Examination)

Die Prüfungen wurden an den Kliniken von 18 % (*n* = 4) der Befragten online (als Take-Home-Klausur) gestaltet. Präsenzprüfungen mit Multiple-Choice-Klausuren wurden bei 46 % (*n* = 10) auf Papier und bei 32 % (*n* = 7) digital durchgeführt. OSCEs (Objective Structured Clinical Examination) fanden bei 14 % (*n* = 3) und praktische Prüfungen bei 5 % (*n* = 1) der Befragten statt. Außerdem gab ein Befragter (entsprechend 5 %) an, Prüfungen in Form benoteter Online-Hausaufgaben durchgeführt zu haben (Abb. 3).

Befragt wurden die Teilnehmer auch zur Bewertung ihrer Lehre vor und im Wintersemester 2020/21. Dabei bewerteten vor dem Wintersemester 9 % (*n* = 2) ihre Lehre selbst als sehr gut, 73 % (*n* = 16) als gut, 14 % (*n* = 3) als befriedigend und 5 % (*n* = 1) als ausreichend. Die Studierenden hätten nach Angaben der Befragten die Lehre vor dem Wintersemester zu 18 % (*n* = 4) als sehr gut, 50 % (*n* = 11) als gut und 5 % (*n* = 1) als befriedigend bewertet; 27 % (*n* = 6) der Befragten machten keine Angabe zur Bewertung durch die Studierenden.

Befragt zur Einschätzung ihrer Lehre im Wintersemester 2020/21, gaben 9 % (*n* = 2) ihre Lehre als sehr gut, 68 % (*n* = 15) als gut, 23 % (*n* = 5) als befriedigend an. Die Studierenden hätten wieder nach Angaben der Befragten die Lehre im Wintersemester zu 18 % (*n* = 4) als sehr gut, 41 % (*n* = 9) als gut und 5 % (*n* = 1) als befriedigend bewertet; 36 % (*n* = 8) der Befragten machten keine Angabe zur Bewertung durch die Studierenden.

### Befragung Lehrender an der Universitätsmedizin Mainz

**Abb. 4:**
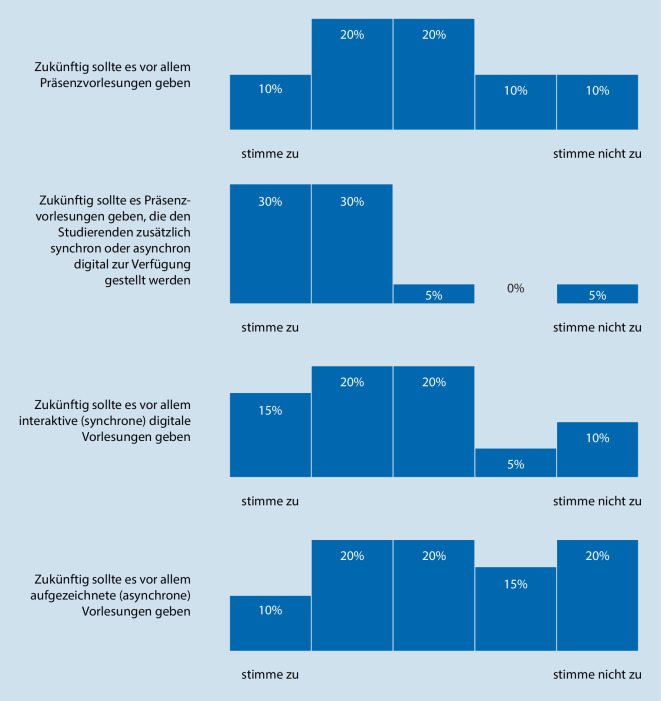
Darstellung der Einschätzung zu digitalen Vorlesungen in der Umfrage unter den befragten ärztlichen Mitarbeitern in Mainz

In unserer internen Befragung der an der Lehre beteiligten Ärzte an der Augenklinik der Universitätsmedizin Mainz befragten wir die Teilnehmer zu ihrer Einschätzung der digitalen Vorlesungen (Abb. 4). Dabei gaben 100 % (*n* = 14) der Befragten an, dass sich mit aufgezeichneten Vorlesungen kognitive Lernziele abbilden lassen, weiterhin gaben 14 % (*n* = 2) affektive Lernziele und 7 % (*n* = 1) psychomotorische Lernziele an.

Befragt zur Einschätzung zur Zukunft von Vorlesungen als Säule der studentischen Lehre stimmten die meisten Befragten einem Konzept aus Präsenzvorlesungen zu, die den Studierenden in der Folge auch digital zur Verfügung stehen sollten. Reine asynchrone Vorlesungen ohne Interaktionsmöglichkeit fanden die geringste Zustimmung.

Online-Videoseminare möchten 86 % (*n* = 12) der Teilnehmer unserer internen Mainzer Befragung in „Post-Corona“-Zeiten weiterhin anbieten. Dabei gaben 79 % (*n* = 11) der Befragten an, dass sich mit Online-Videoseminaren kognitive Lernziele abbilden lassen, weiterhin gaben 36 % (*n* = 5) affektive Lernziele und 14 % (*n* = 2) psychomotorische Lernziele an.

Es können sich 86 % (*n* = 12) der Befragten aus Mainz vorstellen, zukünftig digitale Lehre in ihr reguläres Lehrkonzept zu implementieren. Die digitalen Lehrformate, die dabei eingesetzt werden sollen, können Tab. 3 entnommen werden.

**Tab. 3:** Vorstellungen zur Anwendung digitaler Lehrformate in Mainz

Digitale Lehrformate	Zukünftige Anwendung *n* = 14 Fragebögen, Häufigkeit der Antwort in Prozent (Anzahl)
*Online-Videoseminare*	
Interaktiv	57 (*n* = 8)
Nicht interaktiv	7 (*n* = 1)
*Livestreams von Vorlesungen*	57 (*n* = 8)
*Aufgezeichnete Vorlesungen*	
Ohne Interaktion	43 (*n* = 6)
Mit Interaktionsmöglichkeit (z. B. Quizfragen)	79 (*n* = 11)
*Aufgezeichnete Videos zu unterschiedlichen Techniken und Vorgängen (Spaltlampenuntersuchung, Konfrontationsperimetrie etc.)*	79 (*n* = 11)
*Live-Patientendemonstration im Rahmen eines „virtuellen Patientenzimmers“*	57 (*n* = 8)

## Diskussion

Unsere Befragungen hatten das Ziel, die Sichtweisen der Lehrbeauftragten und maßgeblich an der studentischen Lehre beteiligten, ärztlichen Mitarbeiter der Augenheilkunde zur studentischen Lehre während des coronabedingt eingeschränkten Wintersemesters 2020/21 zu untersuchen. Dabei konnten wir feststellen, dass digitale Lehrkonzepte im Wintersemester 2020/21 eine breite Anwendung fanden. In unserer Befragung gab die Mehrheit der Teilnehmer an, dass an ihren Universitätskliniken in der Augenheilkunde seit der Corona-Pandemie digitale Lehre fest in den Lehrplan implementiert wurde. Innerhalb kurzer Zeit wurden an fast allen universitären Augenkliniken parallel Lehrkonzepte mit digitalen Lehrformaten entwickelt, was der eigenen Erfahrung nach initial mit einem hohen zeitlichen Mehraufwand in der Produktion und Aufbereitung verbunden war. An einigen Kliniken waren bereits zuvor digitale Lehrkonzepte etabliert gewesen, z. B. im Rahmen von Modellstudiengängen. Dies war zu Beginn der Corona-Pandemie vorteilhaft gewesen, da Abläufe bereits eingespielt waren.

In der deutschlandweiten Befragung zeigte sich, dass auch für die Zukunft eine Fortsetzung der digitalen Lehre als Ergänzung zur vor der Corona-Pandemie bestehenden Präsenzlehre von den Lehrbeauftragten gewünscht wird. Die große Mehrheit der Lehrbeauftragten möchte Teile der digitalen Lehrformate auch in Zukunft in die regulären Lehrkonzepte implementieren. Dies deckt sich auch mit internationalen Untersuchungen. So wurde in einer Befragung der *International Retina Collaborative *unter Ophthalmologen mit aktiver Beteiligung in der augenheilkundlichen Lehre festgestellt, dass über 80 % der Befragten glauben, dass die während der Pandemie entwickelten Methoden des „E-Learnings“ in der zukünftigen Lehre in der Augenheilkunde weiter angewendet werden [2].

Dabei werden insbesondere ergänzende Formate wie Videos zu Untersuchungstechniken als wertvoll erachtet. Dies deckt sich auch mit den Ergebnissen der Hochschulrektorenkonferenz. Hier wird digitale Lehre als Chance gesehen, didaktische Konzepte zu erweitern [3]. Im Gegensatz dazu möchte die Mehrheit der Befragten die asynchronen Vorlesungen ohne Interaktionsmöglichkeit zukünftig nicht fortsetzen. Dabei könnten asynchrone aufgezeichnete Vorlesungen im Sinne von Impulsreferaten, also kürzere Einheiten, die auf das ergänzende Selbststudium vorbereiten sollen, die Lehrkonzepte um ein zeitlich flexibles Format sinnvoll ergänzen und somit insbesondere auch die Partizipation von Studierenden mit individuellen Lebensverhältnissen fördern [4].

Gleichzeitig besteht ein großer Wunsch zur Rückkehr der Präsenzlehre. Präsenzlehre und direkter Patientenkontakt bleiben nach wie vor Methode der Wahl zur Vermittlung praktischer Fertigkeiten in der Augenheilkunde. Dies bestätigte sich auch in der weltweiten Befragung von Chatziralli et al. [7]. Die digitalen Lehrformate sollen dabei v. a. eine ergänzende Rolle spielen. In Zukunft werden sich vermutlich Lehrkonzepte mit Anwesenheits- und Onlinephasen im Sinne eines „blended learning“ etablieren [1]. Hierbei können kognitive und auch affektive Lernziele anteilig oder vollständig digital gelehrt und die – mit digitalen Medien nur schwer umzusetzenden – psychomotorischen Lernziele präsent an Simulatoren oder im Rahmen eines „bedside teaching“ unterrichtet werden. Simulationen vor dem Unterricht am Krankenbett bieten sich häufig an, um den Studierenden eine gewisse Sicherheit zu geben [5], und lassen sich auch im Bereich der Ophthalmologie gut umsetzen. Das Konzept des „flipped/inverted classroom“ mit initialer eigenständiger Vorbereitung durch E‑Learning und anschließender Diskussion ist ebenfalls eine Methode, die sich in der medizinischen Lehre erfolgreich etabliert [9].

In unserer deutschlandweiten Befragung wurden die Teilnehmer auch zu Möglichkeiten und Herausforderungen der digitalen Lehrformate im Einsatz während der Corona-Pandemie befragt. Trotz der Einschränkungen der Präsenzlehre waren durch verschiedene digitale Lehrformate vielfältige Möglichkeiten zur studentischen Lehre gegeben. So konnten Vorlesungen, Online-Seminare, Untersuchungskurse und Patientendemonstrationen stattfinden.

Dabei galt es jedoch, verschiedene Hürden zu überwinden, um die Studierenden und die gesetzten Lernziele zu erreichen. Insbesondere wurden vielerorts die Interaktion mit den Studierenden und direktes Feedback als erschwert eingeschätzt. Trotz interaktiver Online-Formate, in denen der Austausch via Chat oder direkt im Videoseminar möglich war, wurde weniger Interaktion bemängelt. Hierbei fielen v. a. im Sommersemester 2020, also in vielen Fällen dem ersten Semester mit festem Lehrkonzept unter coronabedingten Kontaktbeschränkungen, reduzierte Mitarbeit und weniger Rückfragen auf. Im letzten Wintersemester 2020/21 konnten bereits eine bessere Annahme und ein vermehrtes Einbringen der Studierenden beobachtet werden. Dabei ist möglicherweise mit bereits etabliertem digitalem Lehrkonzept und entsprechender technischer Ausstattung der Klinik auch eine bessere Akzeptanz der Studierenden vergesellschaftet. Auch international wurden Hürden für digitale Lehrformate mit weniger Interaktion, fehlende technische Voraussetzungen und verminderte Akzeptanz digitaler Lehre konstatiert [2].

Im Rahmen der deutschlandweiten Online-Befragung wurde angegeben, dass die große Mehrheit der deutschen Universitätskliniken während des Wintersemesters 2020/21 v. a. digitale Lehrformate einsetzte. Eine Präsenzlehre war durch die Vorgaben zur Pandemieeindämmung nur in reduziertem Umfang möglich gewesen. Trotzdem wurden in den Prüfungssituationen überwiegend weiterhin Präsenzformate eingesetzt. Dabei wurden mehrheitlich die etablierten Multiple-Choice-Klausuren verwendet, ergänzend auch in Präsenzform praktische Prüfungen, OSCEs und mündliche Prüfungen zur Wissensabfrage eingesetzt. Nur geringe Anwendung fanden hingegen sog. Take-Home-Klausuren, bei denen die Prüfung zu Hause, auch online, durchgeführt werden kann.

## Schlussfolgerung

Die Corona-Pandemie hatte durch die nötigen Kontaktbeschränkungen auch in der Augenheilkunde weitreichende Folgen für die studentische Lehre. So kamen deutschlandweit digitale Lehrkonzepte zum Einsatz. Diese waren z. T. bereits vorher regelhaft in den Lehrbetrieb integriert, wurden z. T. aber auch erst während der „Corona-Semester“ entwickelt. Hierbei wurden an vielen Orten parallel vielfältige Formate ausgetestet. Beobachtungen, die jetzt gemacht werden, können dabei als Wegweiser für die Zukunft dienen. So werden künftig digitale Formate im Lehrbetrieb weiter eine Rolle spielen. Präsenzformate wie interaktive Vorlesungen können dabei durch digitale Angebote unterstützt werden. So können Videos z. B. zu einzelnen Untersuchungen oder Krankheitsbilder über Patientenvorstellungen als Vorbereitung auf den Präsenzunterricht in der Klinik genutzt werden.

Die in der Notsituation der Corona-Pandemie begonnene Entwicklung digitaler Lehrkonzepte kann den Ausgangspunkt für einen Wandel in der studentischen Lehre in der Augenheilkunde bilden. Dabei bleibt das Vermitteln praktischer Lehrinhalte im direkten Patientenkontakt wichtiger Bestandteil. Die Anerkennung der Veränderungen in der studentischen Lehre in der Augenheilkunde bietet die Chance einer modernen Ausbildung künftiger Generationen von Ophthalmologen in Deutschland.
